# Osteomyelitis of the Mandible after Dental Implants in an Immunocompetent Patient

**DOI:** 10.1155/2017/9525893

**Published:** 2017-04-02

**Authors:** Matthieu Balanger, Margaux Hinet, Christian Vacher, Norbert Bellaiche, Jean-Luc Charrier, Sarah Millot

**Affiliations:** ^1^Department of Oral Surgery, Charles Foix Hospital, Paris Descartes University, Paris, France; ^2^Department of Prosthetic Dentistry, Charles Foix Hospital, Paris Descartes University, Paris, France; ^3^Department of Maxillofacial and Stomatology Surgery, Beaujon Hospital, Paris, France; ^4^Radiology, Private Practice, Paris, France; ^5^Department of Oral Surgery, Bretonneau Hospital, Paris Descartes University, Paris, France; ^6^Department of Oral Surgery, Montpellier Hospital, INSERM UMR1149 Xavier Bichat University, Paris, France

## Abstract

Dental implants are now broadly used to replace missing teeth, and the presence of infectious complications is rising. Dental implant therapy as a local risk factor for the onset of osteomyelitis and its management have not been widely explored. Here, we report an unusual case of mandibular suppurative osteomyelitis caused by* Streptococcus intermedius* in a healthy and immunocompetent patient secondary to mandibular implants. We describe how surgery combined with systemic application of antibiotics allowed conservation of the dental implants in the mandibular bone, discuss the probable source of contamination, and present the follow-up of the osteomyelitis.

## 1. Introduction

Osteomyelitis is an infection of the bone that has a tendency to involve the adjacent cortex, periosteum and soft tissue. Osteomyelitis of the jaw is confined to the mandible in most cases, very likely due to the complex anatomy and the poor vasculature of this bone in contrast to the maxilla, which is characterized by rich vascularity and a thin cortical plate [1, 2].

The inflammatory process includes necrosis of mineralized and marrow tissues, resorption, sclerosis, and hyperplasia; surgical debridement in addition to antibiotic therapy is necessary for cure. Osteomyelitis has exhibited a decline in prevalence, which has been attributed to the widespread use of antibiotics and better oral health. Nevertheless, this infection is known to occur in immunocompromised patients and is generally caused by inoculation of microorganisms into the jawbones as a result of trauma, a surgical procedure, dental infection, or chemotherapeutic drug use [3–6].

Despite the increasing popularity of using implants to rehabilitate edentulous alveolar ridges and these implants' high long-term success rate, certain adverse complications (biological, mechanical) can occur. However, to the best of our knowledge, only a few case reports to date have linked infection leading to osteomyelitis of the jaws to dental implant therapy [7–10]. Dental practitioners should recognize the signs of this disease, know how to treat it, and be able to accurately determine whether removal of the implant is necessary for successful management.

## 2. Case Presentation

A healthy female patient, aged 71 years and without medication, was referred to our Department of Oral Medicine with substantial pain and swelling in the submandibular area that had developed after the second stage of a dental treatment protocol.

**Figure 1:**
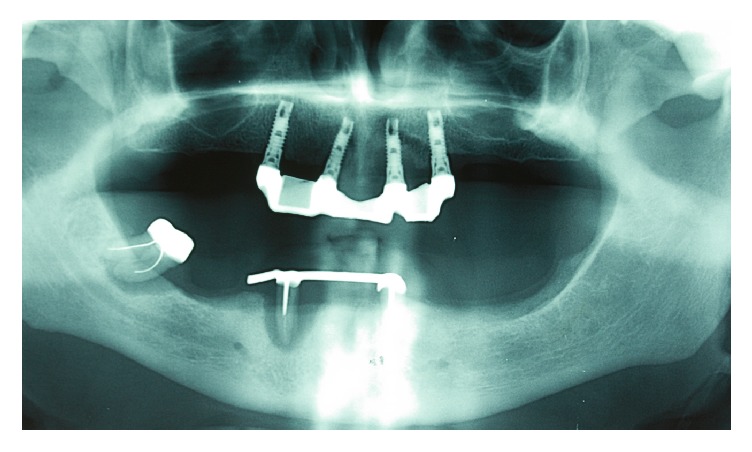
Panoramic radiograph showing periapical lesions related to the right mandibular second molar and canine and the left mandibular canine.

**Figure 2:**
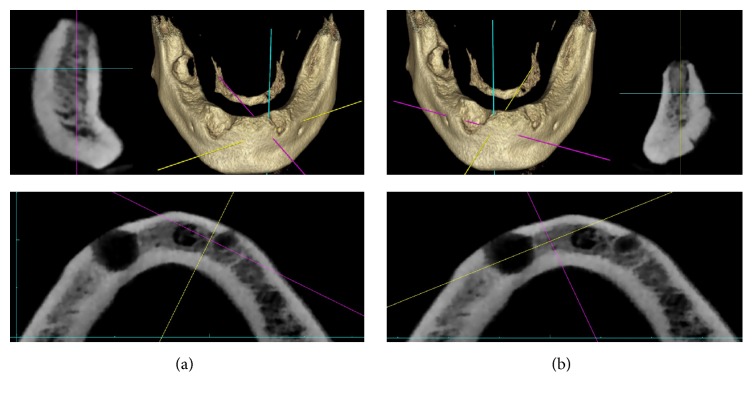
(a) Preoperative CBCT (cross-sectional and axial images) evaluation of the mandibular symphyses after dental extractions. Left mandibular position of the implant. (b) Preoperative CBCT (cross-sectional and axial images) evaluation of the mandibular symphyses after dental extractions. Right mandibular position of the implant.

The patient's past dental history revealed extractions of mandibular right first molar and both mandibular canines due to periapical lesions, with curettage of granulation tissue (Figure 1). Five months after the tooth extractions, an implant-based therapeutic project was proposed, and preoperative cone-beam computed tomography (CBCT) was performed prior to dental implant therapy to analyze the left (Figure 2(a)) and right Figure 2(b) mandibular symphyses. Two osseointegrated dental implants (canine regions) were then placed using a two-stage procedure (Nobel Biocare Speedy Groovy, 4*∗*10 mm) with oral administration of amoxicillin (2 g/per day) for 6 days. The surgery was performed by experienced surgeons in a specific operating room with strict aseptic condition.

The implants were submerged and left in place for a period of three months. Then, the second-stage surgery, which involved placement of prosthetic abutments, was performed without any problem by the same surgical team. Unlike implant surgery, the aseptic conditions were lower but in line with this type of intervention. One week later, the patient was referred to an emergency consultation for facial pain. Clinical examination revealed diffuse swelling of the submandibular region, which was firm and painful, with redness of the skin. The patient also reported labiomandibular paresthesia.

On intraoral examination, there was gingival inflammation in the anterior part of the mandible. The swelling involved both the gingiva and the buccal vestibule.

**Figure 3:**
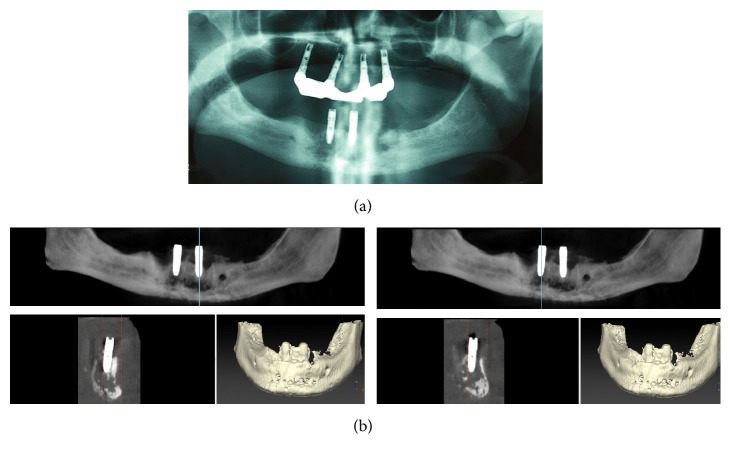
(a) Panoramic radiograph showing bone osteolytic lesions without defined borders after the second-stage surgery. (b) CBCT urgently performed showing osteolysis, with lingual and buccal cortical interruption.

A panoramic radiograph revealed a radiolucent, diffuse osteolytic lesion with poorly defined borders in the symphysis area of the mandible (Figure 3(a)). CBCT was urgently performed to assess the lesion and revealed extensive osteolysis, with destruction of the cortical borders in the area of the implants. Alveolar and basal bones are affected (Figure 3(b)). Based on clinicoradiological findings, a provisional diagnosis of chronic osteomyelitis was made.

On the same day, the patient was transferred to the Department of Oral and Maxillofacial Pathology, and surgical intervention was performed under general anesthesia via an intravenous injection of amoxicillin/clavulanate (1 g/200 mg).

**Figure 4:**
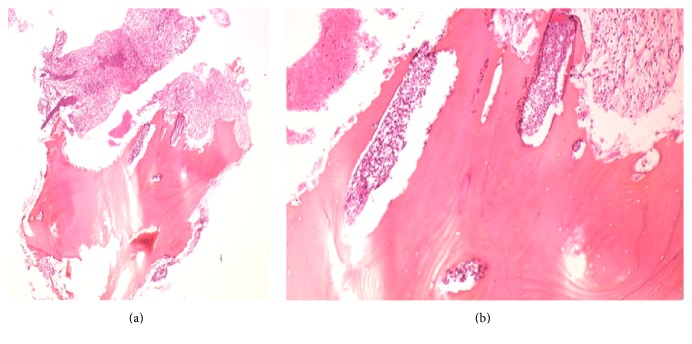
(a) Microscopic view of the bone biopsy. Hematoxylin-eosin staining (HES), low-magnification view (×10). Diffuse neutrophil infiltrate and osteoclastic bone resorption through neutrophil-osteoclast interactions. (b) HES, higher magnification (×40). Intense acute inflammation with numerous neutrophils and images of bone resorption.

The affected mandibular region was opened, and a sequestrectomy of the mental region was performed. The area was thoroughly debrided and irrigated, and the two dental implants were left in place. A bone biopsy was sent for histopathology analyses, which confirmed the diagnosis of chronic osteomyelitis with diffuse neutrophil infiltration (Figures 4(a) and 4(b)). A microbiologist also used cell culture to identify* Streptococcus intermedius *bacteria.

**Figure 5:**
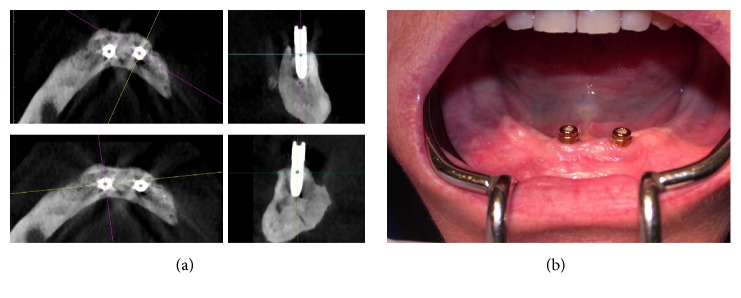
(a) Follow-up after surgery. CBCT images 12 months after osteomyelitis. (b) Intraoral photography showing the two dental implants without inflammation 12 months after osteomyelitis.

The patient was hospitalized, and, five days after surgery, the symptoms and swelling disappeared. An oral course of antibiotics was prescribed for the following 2 months (amoxicillin 1 g two times per day). The follow-up consisted of one control consultation per week for one month and one control consultation per month for six months thereafter. Clinical and radiological analyses revealed healing in the mandibular region and no local complications. There was no evidence of local recurrence at 12 months (Figures 5(a) and 5(b)) after treatment, and placement of prosthetic abutments was performed. Prosthetic treatment was also performed.

## 3. Discussion

Many species of bacteria have been implicated in osteomyelitis, but most described pathogens belong to the family of staphylococci [11].* Streptococcus intermedius* was the cause of the osteomyelitis in the patient described here; this bacterial strain belongs to the* Streptococcus anginosus* group. This group is recognized as consisting of commensal bacteria of the oral cavity and the gastrointestinal and urogenital tracts but is also known for abscess formation in various locations in the body and for infective endocarditis [12].

In the current case, the patient was immunocompetent and lacked medical risk factors, and the patient's osteomyelitis was caused by a bacterial species that is not known to have a tropism for bone. Griffin et al. published the largest series of patients with osteomyelitis due to* Streptococcus anginosus* group bacteria, including 297 patients with osteomyelitis, and only eleven patients with* Streptococcus anginosus* organisms were identified. Among these, three cases of mandibular osteomyelitis were associated with tooth decay and bisphosphonate or radiation exposure [13].

In the current study, the infection was most likely related to contamination of dental implants. The first-stage surgery occurred in a clinical setting dedicated to implantology and was without complications. Three months afterward, the second-stage surgery was performed with placement of prosthetic abutments, and, one week later, the infection was diagnosed due to pain and swelling. We cannot rule out the possibility that periapical lesions of teeth were the source of bacterial contamination and infection before implant insertion, but the absence of early signs is remarkable, and the dental implants were placed several months afterward, allowing a long time for bone healing. During surgery, after curettages of nonvital bone, a conservative approach was selected, and the two implants were left in place. However, there is limited literature on the guidelines for removing or leaving implants in place if osteomyelitis is diagnosed. The fundamental principles of the treatment of osteomyelitis remain debridement and antibiotic therapy, but some new approach using vancomycin-impregnated calcium sulfate in the surgical debridement site seems to give good results [14]. Indeed, this therapy maintains an effective topical antibiotic concentration for a long time by sustaining the release of antibiotics and calcium sulfate has bone conduction and potential bone-inducing effects that can promote the formation of new bone [14].

Implant removal is recommended when apical lesions are involved and when mobility of the implants is confirmed [15].

Given the popularity of an increase in dental implant treatments, it seems necessary to discuss the failure and complications of this treatment. Conventional protocols for endosseous implants (these dental implants are kept load free for three months) were shown to minimize the risk of implant failure and to establish osseointegration, with a high rate of success. Nevertheless, recently, Camps-Font et al. in a retrospective cohort study with 1273 implants included in 337 patients highlighted that 4 to 10% of patients receiving dental implants develop postoperative infections and that two-thirds of infected implants fail, most before prosthetic loading [16]. To address functional and aesthetic requirements, less time-consuming techniques are increasingly available. Indeed, immediate loading after tooth extraction via either the installation of implants into postextraction sockets without time for healing or the installation of implants into infected sites has been proposed [17, 18].

We believe that this approach should be measured and used with caution especially in infected sites and those even in absence of clinical symptoms considering that it could induce potentially severe bone infections. It is challenging for clinicians to provide rapidly effective treatments with fewer complications to maintain the health of the alveolar bone [19, 20].

## 4. Conclusion

Implant surgery has become widespread but remains an invasive bone surgery that can lead to serious infectious complications, including osteomyelitis. Strict aseptic operating conditions and a healthy oral environment are recommended. The causative organisms may be commensals in healthy individuals, as we have presented in this case. Physicians have to identify infectious implant complications and their etiology to make implant treatment even more predictable in the future.
